# Alterations of the endocannabinoid system and circulating and peripheral tissue levels of endocannabinoids in sarcopenic rats

**DOI:** 10.1002/jcsm.12855

**Published:** 2021-12-02

**Authors:** Olivier Le Bacquer, Jérôme Salles, Fabiana Piscitelli, Phelipe Sanchez, Vincent Martin, Christophe Montaurier, Vincenzo Di Marzo, Stéphane Walrand

**Affiliations:** ^1^ INRAE, Unité de Nutrition Humaine (UNH) Université Clermont Auvergne Clermont‐Ferrand France; ^2^ Institute of Biomolecular Chemistry National Research Council Pozzuoli Italy; ^3^ AME2P Université Clermont Auvergne Clermont‐Ferrand France; ^4^ Institut Universitaire de France (IUF) Paris France; ^5^ Canada Excellence Research Chair Microbiome‐Endocannabinoidome Axis in Metabolic Health, Faculty of Medicine and Faculty of Agricutural and Food Sciences, IUCPQ, INAF and Centre NUTRISS Université Laval Quebec City Quebec Canada; ^6^ CHU Clermont‐Ferrand, Service de Nutrition Clinique Hôpital Gabriel Montpied Clermont‐Ferrand France

**Keywords:** Skeletal muscle, Muscle function, Sarcopenia, Endocannabinoids

## Abstract

**Background:**

Activation of the endocannabinoid system (ECS) is associated with the development of obesity and insulin resistance, and with perturbed skeletal muscle development. Age‐related sarcopenia is a progressive and generalized skeletal muscle disorder involving an accelerated loss of muscle mass and function, with changes in skeletal muscle protein homeostasis due to lipid accumulation and anabolic resistance. Hence, both obesity and sarcopenia share a common set of pathophysiological alterations leading to skeletal muscle impairment. The aim of this study was to characterize how sarcopenia impacts the ECS and if these modifications were related to the loss of muscle mass and function associated with aging in rats.

**Methods:**

Six‐month‐old and 24‐month‐old male rats were used to measure the contractile properties of the plantarflexors (isometric torque–frequency relationship & concentric power–velocity relationship) and to evaluate locomotor activity, motor coordination, and voluntary gait by open field, rotarod, and catwalk tests, respectively. Levels of endocannabinoids (AEA & 2‐AG) and endocannabinoid‐like molecules (OEA & PEA) were measured by LCF‐MS/MS in plasma, skeletal muscle, and adipose tissue, while the expression of genes coding for the ECS were investigated by quantitative reverse transcription PCR (RT‐qPCR).

**Results:**

Sarcopenia in old rats was exemplified by a 49% decrease in hindlimb muscle mass (*P* < 0.01), which was associated with severe impairment of isometric torque, power, voluntary locomotor activity, motor coordination, and gait quality. Sarcopenia was associated with (1) increased 2‐AG (+32%, *P* = 0.07) and reduced PEA and OEA levels in the plasma (−25% and −40%, respectively, *P* < 0.01); (2) an increased content of AEA, PEA, and OEA in subcutaneous adipose tissue (*P* < 0.01); and (3) a four‐fold increase of 2‐AG content in the soleus (*P* < 0.01) and a reduced OEA content in EDL (−80%, *P* < 0.01). These alterations were associated with profound modifications in the expression of the ECS genes in the adipose tissue and skeletal muscle.

**Conclusions:**

Taken together, these findings demonstrate that circulating and peripheral tissue endocannabinoid tone are altered in sarcopenia. They also demonstrate that OEA plasma levels are associated with skeletal muscle function and loss of locomotor activity in rats, suggesting OEA could be used as a circulating biomarker for sarcopenia.

## Introduction

Age‐related sarcopenia is a progressive and generalized skeletal muscle disorder involving an accelerated loss of muscle mass and function without any underlying disease. In older individuals, depending on the degree of intensity of these changes, alterations of the locomotor system could lead to decreased physical functions and increased frailty, which progressively leads to impaired mobility, loss of independence, and increased mortality.^1^ With increasing life expectancy and population, there is no doubt that the prevalence of sarcopenia will increase in the coming decades. The detailed understanding of the molecular mechanisms contributing to sarcopenia is therefore of growing importance in order to develop efficient preventive and therapeutic strategies.

The pathogenesis of sarcopenia is complex, and many mechanisms are believed to contribute to its development. Several molecular mechanisms have been described as causes for sarcopenia that refer to very different levels of muscle physiology. These mechanisms cover quantitative and qualitative changes in skeletal muscle structure, decreased contractility capacity, impaired control of muscle contraction by the nervous system, impaired regenerative potential, and changes in the regulation of protein metabolism driven by nutrients and hormones. This later mechanism is characterized by an altered anabolic and anti‐catabolic action of hormones (insulin, IGF‐1 …)^2^ and nutrients (amino acids).^3^ Mitochondrial dysfunctions^4^ that are partly due to an increased accumulation of lipids within the skeletal muscle also contribute to sarcopenia.^5^ During obesity, after adipocytes exceed their storage capacity, lipids begin to accumulate in non‐adipose tissues (e.g. liver & skeletal muscle) where they induce the production of toxic lipid derivatives such as ceramides, ultimately culminating in insulin resistance,^6^ and muscle wasting.^7^ Therefore, both obesity and sarcopenia share a common set of pathophysiological alterations leading to skeletal muscle impairment.

A large body of evidence demonstrates that excessive nutritional intakes overactivate the endocannabinoid system, which favours the development of obesity and metabolic diseases.^8^ Anandamide (*N*‐arachidonoylethanolamine, AEA) and 2‐AG (2‐arachidonoylglycerol) are the most extensively studied members of *N*‐acylethanolamines (NAE) and monoacylglycerol families, respectively. Together with the enzymatic machinery responsible for their metabolism and at least two G‐protein coupled receptors, the cannabinoid type‐1 (CB1) and type‐2 (CB2) receptors, they define the endocannabinoid system (ECS).^8^ Other endogenous ligands are called endocannabinoid‐like compounds, because they share this enzymatic system, but are unable to efficiently activate cannabinoid receptors. Among these compounds are NAEs such as *N*‐oleoylethanolamide (OEA) and *N*‐palmitoylethanolamine (PEA), which modulate lipid metabolism and exhibit anti‐inflammatory properties, respectively.^9^ In the last decade, studies highlighted the role of the ECS in the control of skeletal muscle metabolism and development. *In vitro*, the treatment of skeletal muscle cells with an adipocyte‐conditioned medium containing AEA and 2‐AG or with ACEA (a synthetic CB1 agonist) reduces, while treatment with rimonabant (a CB1 antagonist) improved glucose uptake^10^, ^11^, ^12^ by modulating insulin sensitivity.^10^, ^11^ In addition to controlling skeletal muscle metabolism, endocannabinoids are also involved in skeletal muscle development and dysfunction.^13^, ^14^ In a first study, Iannotti *et al*. observed that 2‐AG levels decreased during myotube differentiation from C2C12 myoblasts and mouse muscle development. They also demonstrated, *in vitro*, that direct activation of CB1 by ACEA or 2‐AG was able to prevent myotube formation from myoblast and to promote myoblast proliferation.^13^ Overexpression of CB1 mRNA was observed in both satellite cells and skeletal muscle of mdx mice (a model of Duchenne's muscular dystrophy) concurrently to the first signs of the disease.^14^ The treatment of satellite cells with rimonabant increased their differentiation and the number of regenerated myofibres, and prevented the loss of mobility in mdx mice.^14^ Last, in a recent study, we demonstrated that acute inhibition of the ECS by rimonabant was able to prevent dexamethasone‐induced atrophy and to stimulate protein synthesis in a model of myotubes in culture.^15^

Whether sarcopenia is associated with modifications of endocannabinoid tone and alterations of the ECS is, to our knowledge, unknown. Therefore, the aim of the present study was (1) to characterize the changes of the circulating and tissue levels of endocannabinoids in a context of sarcopenia, (2) to characterize the alterations of the ECS genes in the adipose tissue and skeletal muscle, and (3) to analyse whether these alterations can be associated with muscle mass, function, and mobility in old rats.

## Methods

### Animals and ethical

All experimental protocols were reviewed and approved by the local ethics committee for animal experimentation (CREFA Auvergne, agreement # 21250‐2019062711554233) and met the National Research Council's guideline for the care and use of laboratory animals. Six‐month‐old and 24‐month‐old male Wistar rats were purchased from Janvier Laboratory (Saint Bertevin, France). Rats were individually housed in plastic cages and maintained at 21–23°C with a 12:12 h light–dark schedule, given free access to water and food. Rats were fed a normal chow diet (25% protein, 61% carbohydrate, and 14% fat, Safe‐Diets, Augy, France). At the end of the mobility and skeletal muscle function analyses, skeletal muscles and adipose tissues were quickly removed from animal anaesthetised by isoflurane inhalation. Samples were weighed, immediately frozen in liquid nitrogen, and stored at −80°C until analyses. Blood samples were collected from the abdominal aorta and drawn into precooled ethylenediaminetetraacetic tubes. These tubes were centrifuged, and plasma was removed and frozen at −80°C until analysis. Euthanasia was performed at 9:00 AM after an overnight fasting where rats had free access to water.

### Indirect calorimetry and whole body composition analysis

Energy expenditure, food, and water intakes were measured by using a PhenoMaster/LabMaster four‐cage TSE system (Bad Homburg, Germany). For whole body composition analysis, rats were placed in an EchoMRI‐100 analyser (Echo Medical Systems LLC, Houston, TX) to determine fat and lean body mass (g).

### Catwalk test

Gait analysis was performed using the CatWalk XT™ system (Noldus Information Technology, Netherlands). The CatWalk XT™ system is a complete automated and highly sensitive tool to assess voluntary gait and locomotion. It can be used to evaluate any kind of experimental model or procedure that has an effect on the locomotor ability of small animals (i.e. rat & mouse). Similar to clinical gait tests in humans, this system allows rodent to voluntarily move at a preferred speed. The device consists of a 130 cm long hardened glass platform with an adjustable alleyway to limit movements to straight lines, a green LED light attached to the glass platform, a high‐speed colour camera mounted below the platform, and a goal‐box at the end containing the home cage of the animals. The green LED light attached to the apparatus emits light into the glass plate, and this light is only refracted wherever rodent paws contact the glass, allowing the high‐speed digital camera to capture precise rodent paw placement in real time. To perform the gait analysis, each rat was trained to be able to cross the runway set to a width of 11 cm. The high‐speed video camera was fixed 70 cm below the corridor to allow the record of five full step cycles. For each animal, three compliant runs were acquired. Compliant runs were defined as runs spending longer than 0.50 s, but shorter than 7.0 s, with a maximum allowed speed variation ≤ 40%. Semi‐automated labelling and analysis of paw prints with CatWalk XT™ software provided static and dynamic gait parameters as follows:

- *Average gait speed* (cm/s): average speed of a run.
- *Number of steps*: number of steps to perform the run.
- *Cadence* (steps/s): number of steps by second.
- *Stand* (s): duration of rat paw contact with the glass plate.
- *Swing speed mean* (cm/s): time duration without rat paw contact with the glass platform.
- *Stride length mean* (cm): distance between two placements of the same foot.
- *Step cycle* (s): time between two consecutive initial contacts of the same foot.

### Rotarod test

Motor coordination and performance were assessed by using the accelerating rotarod test. Rats were trained to walk on the rod with constant low‐speed rotation (4 rpm) for 3 days with one trial per day (LE‐8355, BIOSEB, Vitrolles, France). For the accelerating test (4–40 rpm over 600 s), three trials per test were performed during the test day, with a 20 min interval between trials. The time until the rat fell from the rod was recorded automatically using the Sedacom software (BIOSEB, Vitrolles, France).

### Open field test

Open field tests were performed to allow the evaluation of animal's general exploratory locomotion in a novel environment. The open field apparatus consisted of an arena made with durable material non‐absorbent to the odours (90 cm × 90 cm and 40 cm high) (BIOSEB, Vitrolles, France). The floor of the arena was divided into three zones. The rats were gently placed individually in a corner of the arena and allowed to explore it freely. For each rat, the locomotor activity was evaluated by measuring total travelled distance (cm), average speed (cm/s), and the duration of activity over a 10 min test period that was recorded using a video tracking camera. SMART Video Tracking software was used to automatically analyse rat movement. The arena was cleaned with cotton soaked in 70% alcohol between each rat test.

### Torque and power tests

The functional properties of the plantarflexor muscles were evaluated *in vivo* on an isokinetic dynamometer specially designed for rats (806D, Aurora Scientific, Canada). Rats were maintained anaesthetised with continuous isoflurane inhalation. During the testing procedures, the rat laid supine on a heating plate with the right foot attached to a footplate connected to a dual mode servomotor (305C‐LR, Aurora Scientific, Canada). The knee was clamped in place such that the knee angle was 90°, and the ankle axis of rotation coincided with axis of the motor. To avoid any variation in body temperature, the rectal temperature was monitored and computed by a temperature controller (ATC 1000, World Precision Instruments, USA) that adjusted the temperature of the heating plate to maintain the rectal temperature at 37°C. Stimulation of the rat ankle plantarflexors was done percutaneously via Ag/AgCl surface electrodes (StimCom TS0020, Comepa, France). A constant‐current electrical stimulator (DS 7A, Digitimer, United Kingdom) was used to deliver square waves (pulse width = 1 ms). The stimulator was triggered and controlled with automated scripts by an A/D board (Powerlab 8/35, ADInstruments, Australia) and associated software (LabChart 7.3, ADInstruments, Australia). The protocol consisted in the evaluation of the torque–frequency relationship in isometric condition and the torque–velocity relationship in concentric condition. To determine the torque–frequency curve, the ankle angle was set at 90°, and the plantarflexors stimulated at frequencies varying from 10 to 200 Hz (10, 20, 30, 40, 50, 60, 70, 80, 100, 125, 150, 175, and 200 Hz). These contractions were 200 ms in length with 45 s between contractions and done in order of increasing stimulation frequency. The reported isometric torque values were calculated as the peak isometric torque minus resting torque. The maximal and mean torque were determined from the torque–frequency relationship. The maximal and mean relative torque were calculated as the torque divided by the muscle mass of the plantarflexor muscles. After 3 min of rest, the torque–velocity curve was determined from 11 concentric contractions realized at angular velocities of 50, 100, 200, 300, 400, 500, 600, 700, 800, 900, and 1000°/s, realized in order of decreasing velocity. These contractions were evoked over a 40°‐angular amplitude, centred about the 90° ankle angle (i.e. from 70° to 110°). This movement range was chosen because it coincides closely to that of the ankle during the stance phase of voluntary ambulation (i.e. from 72° to 111°). The contractions were evoked every 45 s by stimulation trains delivered at 175 Hz for only the duration necessary to complete the movement (i.e. 40 ms at 1000°/s to 800 ms at 50°/s); the 175 Hz frequency was used according to Warren *et al*.^16^ recommendation, as the frequency yielding maximal isometric tetanic torque. A power–velocity curve was then computed from this torque–velocity curve to determine the maximal power. The maximal and mean power as well as the optimal velocity (i.e. the velocity corresponding to maximal power) were determined from the power–velocity relationship. The maximal and mean relative power were calculated as the power divided by the muscle mass of the plantarflexor muscles. Data were recorded with an A/D Board (Powerlab 8/35, ADInstruments, Australia), sampled at a frequency of 2000 Hz and analysed with the Labchart software (LabChart 7.3, ADInstruments, Australia). This software and the Powerlab system were also used to trigger and control the movement of the isokinetic ergometer with automated scripts during concentric contractions.

### Plasma analyses

Overnight fasting insulin level was measured by ELISA (EuroBio, Courtaboeuf, France). Plasma levels of fasting glucose, triglycerides, non‐esterified fatty acids, and cholesterol were determined using a Konelab 20 analyser (Thermo‐Electron Corporation).

### Measurement of endocannabinoids

The extraction, purification, and quantification of EC from tissues have been performed as previously described.^17^ Briefly, tissues were dounce‐homogenized and extracted with chloroform/methanol/Tris–HCl 50 mmol/L pH 7.5 (2:1:1, vol/vol) containing internal standards ([^2^H]^8^ AEA 10 pmol; [^2^H]^5^ 2‐AG, [^2^H]^5^ PEA and [^2^H]^4^ OEA 50 pmol each). The lipid‐containing organic phase was dried down, weighed, and pre‐purified by open‐bed chromatography on silica gel. Fractions were obtained by eluting the column with 99:1, 90:10 and 50:50 (v/v) chloroform/methanol. The 90:10 fraction was used for AEA, 2‐AG, PEA, and OEA quantification by liquid chromatography–atmospheric pressure chemical ionization–mass spectrometry by using a Shimadzu high‐performance liquid chromatography apparatus (LC‐10ADVP) coupled to a Shimadzu (LCMS‐2020) quadrupole mass spectrometry via a Shimadzu atmospheric pressure chemical ionization interface as previously described. LC analysis was performed in the isocratic mode using a Discovery C18 column (15 cm × 4.6 mm, 5 μm) and methanol/water/acetic acid (85:15:1 by vol) as mobile phase with a flow rate of 1 mL/min. The amounts of EC in tissues, quantified by isotope dilution with the abovementioned deuterated standards, are expressed as pmol per mg of tissue weight or mL of plasma volume.

### RNA extraction and quantitative real‐time PCR

Total RNA was extracted using Trizol reagent (Invitrogen) according to the manufacturer's instructions. RNA was quantified by measuring optical density at 260 nm. The concentrations of the mRNAs corresponding to genes of interest were measured by reverse transcription followed by real‐time PCR using a Rotor‐Gene Q (Qiagen) system. One microgram of total RNA was reverse‐transcribed using SuperScript® III reverse transcriptase and a combination of random hexamer and oligo‐dT primers (Invitrogen). PCR amplification was performed in a 20 μL total reaction volume. The real‐time‐PCR mixture contained 5 μL of diluted cDNA template, 10 μL of 2 × Rotor‐Gene SYBR Green PCR master mix, and 0.5 μM of forward and reverse primers. The amplification profile was initiated by 5 min incubation at 95°C to activate HotStarTaq Plus DNA Polymerase, followed by 40 cycles of two steps: 95°C for 5 s (denaturation step) and 60°C for 10 s (annealing/extension step). Relative mRNA concentrations were analysed using Rotor‐Gene software. The relative abundance of mRNAs was calculated using the 2^‐ΔΔCT^ method with HPRT (hypoxanthine–guanine phosphoribosyltransferase) as housekeeping gene for adipose tissue samples and UBC (Ubiquitin C) for skeletal muscle samples. The primers used in the PCR are described in Supporting information, *Table* 1.

**Table 1:** Body composition and hindlimb skeletal muscle and adipose tissue weight in adult and old rats

	Adult	Old
Body weight (g)	590.3 ± 16.7	565.7 ± 20.8
Fat mass (g)	83.7 ± 5.2	108.3 ± 7.9**
Fat mass (%/body weight)	14.1 ± 0.6	19.0 ± 0.9**
Lean mass (g)	466.1 ± 13.25	410.2 ± 13.41**
Lean mass (%/body weight)	79.0 ± 0.7	72.6 ± 0.7**
Soleus (mg)	266.2 ± 6.8	145.4 ± 12.5**
EDL (mg)	235.9 ± 5.4	157.8 ± 10.5**
Tibialis (mg)	892.6 ± 20.6	495.0 ± 40.6**
Plantaris (mg)	526.0 ± 12.1	294.9 ± 21.4**
Gastrocnemius (g)	2.57 ± 0.06	1.24 ± 0.09**
GWAT (g)	6.56 ± 0.35	6.57 ± 0.85
SWAT (g)	6.78 ± 0.46	7.98 ± 0.71

EDL, extensor digitorum longus; GWAT, gonadal white adipose tissue; SWAT, subcutaneous white adipose tissue.

Adult rats and old rats were respectively 6 and 24 months old. Results are expressed as mean ± SEM (*n* = 8–11). *P* values were assessed using unpaired *t*‐test.

* *P* < 0.05,

** *P* < 0.01 vs. adult.

### Western blot analysis

Tissues were homogenized in ice‐cold buffer (50 mM HEPES pH 7.4, 150 mM NaCl, 10 mM ethylenediaminetetraacetic, 10 mM NaPPi, 25 mM β‐glycerophosphate, 100 mM NaF, 2 mM Na orthovanadate, 10% glycerol, & 1% Triton X‐100) containing 1% of a protease inhibitor cocktail (Sigma‐Aldrich, Saint‐Quentin‐Fallavier, France). Homogenates were centrifuged at 13 000*g* for 10 min at 4°C. Denatured proteins were separated by SDS‐PAGE and transferred to a PVDF membrane (Millipore, Molsheim, France). Immunoblots were blocked with TBS‐Tween‐20 0.1% containing 5% dry milk and then probed overnight at 4°C with primary antibodies. After several washes with TBS‐Tween‐20 0.1%, the immunoblots were incubated with a horseradish peroxidase‐conjugated secondary antibody (DAKO, Trappes, France) for 1 h at room temperature. The immune reactive strips or whole lanes were visualized by chemiluminescence (ECL western blotting substrate, Thermo Fisher Scientific, Courtaboeuf, France). Luminescent secondary antibodies were visualized using an MF ChemiBis 2.0 camera (Fusion Solo, Vilber Lourmat, France). Band densities were quantified using the MultiGauge 3.2 software (Fujifilm Corporation, FSVT, Courbevoie, France). Primary antibodies were obtained from the following sources: MAGL antibody was purchased from Abcam (Amsterdam, Netherlands). Total P38 and FAAH antibodies were from Sigma‐Aldrich (Saint‐Quentin Fallavier, France). CB1 antibody was donated by Dr Kenneth Mackie (Indiana University). Horseradish peroxidase‐conjugated secondary antibodies were from DAKO (Trappes, France).

### Statistical analyses

Data are shown as mean ± SEM. Differences between groups (adult vs. old rats) were analysed with a Welch's two‐sample unpaired *t*‐test (Prism 6.05; GraphPad Software Inc. San Diego, USA). Torque–frequency and power–velocity relationships were analysed with a two‐way ANOVA with repeated measures (frequency × group and velocity × group, respectively) after having checked the homogeneity of variances (Levene's test) and sphericity (Mauchly's test). Post‐hoc comparisons were conducted with the Tukey's test. Correlations between endocannabinoid plasma levels and parameters of mobility were analysed by Pearson's correlation tests (NCSS^10^; NCSS, LLC, USA). Statistical significance was set at *P* < 0.05 for all analyses.

## Results

### Analysis of body composition, muscle mass/function, and contractile properties in adult and old rats

Body weight was similar between adult and old rats (*Table* 1). Food intake (26.4 ± 0.8 vs. 23.4 ± 0.7 g/day, *P* < 0.05) and energy expenditure (0.133 ± 0.001 vs. 0.109 ± 0.002 kJ/g of body weight, *P* < 0.01) were reduced in old rats compared with adult rats. Overnight‐fasted glucose (1.39 ± 0.08 vs. 1.48 ± 0.07 g/L) and insulin (0.79 ± 0.17 vs. 1.21 ± 0.22 ng/mL) levels were unchanged between adult and old rats (*Table* 2). Because aging is associated with increased adiposity and loss of muscle mass (sarcopenia), we measured body composition by EchoMRI and the mass of several hindlimb muscles of 6‐month‐old and 24‐month‐old rats. As expected, we observed that fat mass content was higher, and lean mass content was reduced in old rats compared with adult rats (*Table* 1, *P* < 0.01). Analysis of the hindlimb muscles revealed a decrease in the weight of all the muscles collected from old rats compared with adults, confirming that old rats were indeed sarcopenic (*Table* 1, *P* < 0.01). However, no statistical difference was observed in weight for gonadal and subcutaneous adipose tissue (*Table* 1).

**Table 2:** Endocannabinoid concentrations in plasma, skeletal muscles, and adipose tissues of adult and old rats

	Adult	Old
Plasma		
AEA (pmol/mL)	1.94 ± 0.21	2.15 ± 0.32
2‐AG (pmol/mL)	48.88 ± 6.15	64.56 ± 8.34
PEA (pmol/mL)	49.78 ± 5.96	37.11 ± 2.64*
OEA (pmol/mL)	21.40 ± 2.63	12.88 ± 1.10**
Soleus		
AEA (pmol/g)	4.86 ± 0.82	3.20 ± 0.76
2‐AG (pmol/mg)	0.60 ± 0.27	2.44 ± 0.33**
PEA (pmol/mg)	0.19 ± 0.01	0.18 ± 0.03
OEA (pmol/mg)	0.10 ± 0.01	0.09 ± 0.02
EDL		
AEA (pmol/g)	1.72 ± 0.42	1.10 ± 0.50
2‐AG (pmol/mg)	2.32 ± 0.44	2.90 ± 0.31
PEA (pmol/mg)	0.18 ± 0.02	0.16 ± 0.02
OEA (pmol/mg)	0.08 ± 0.01	0.02 ± 0.01**
GWAT		
AEA (pmol/g)	56.03 ± 3.47	58.41 ± 4.98
2‐AG (pmol/mg)	1.98 ± 0.78	2.73 ± 0.72
PEA (pmol/mg)	1.00 ± 0.07	0.90 ± 0.05
OEA (pmol/mg)	0.81 ± 0.06	0.74 ± 0.02
SWAT		
AEA (pmol/g)	36.22 ± 2.67	45.75 ± 2.47*
2‐AG (pmol/mg)	7.74 ± 2.47	3.45 ± 1.93
PEA (pmol/mg)	0.84 ± 0.03	0.95 ± 0.03*
OEA (pmol/mg)	0.73 ± 0.03	1.00 ± 0.03**

2‐AG, 2‐arachidonyl glycerol; AEA, anandamide; EDL, extensor digitorum longus; GWAT, gonadal white adipose tissue; OEA, oleoylethanolamide; PEA, palmitoylethanolamide; SWAT, subcutaneous white adipose tissue.

Adult rats and old rats were respectively 6 and 24 months old. Results are expressed as mean ± SEM (*n* = 8–11). *P* values were assessed using unpaired *t*‐test.

* *P* < 0.05,

** *P* < 0.01 vs. adult.

**Figure 1:**
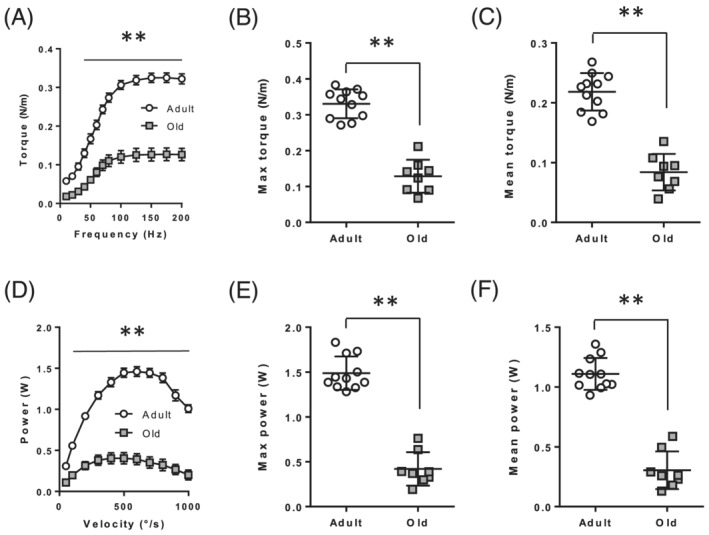
Contractile properties of the plantarflexor are impaired in old rats. (*A*) Absolute torque–frequency relationship in adult and old rats. (*B*) Maximal absolute torque. (*C*) Mean absolute torque. (*D*) Absolute power–frequency relationship in adult and old rats. (*E*) Maximal absolute power. (*F*) Mean absolute power. Values are expressed as mean ± SEM. *P* values were assessed by two‐way ANOVA and Tukey post‐test as described in Method (in *A* & *D*), or by unpaired *t*‐test (in *B*, *C*, *E*, & *F*). **P < 0.01 vs. adult.

Muscle contractile properties were also altered in old rats compared with adult rats (*Figure* 1). Torque was reduced from 40 (*P* < 0.01) to 200 Hz (*P* < 0.001), such that the maximal and mean torque were significantly reduced in old compared with adults (*Figure* 1A–C). The relative torque values, that is, torque expressed per unit muscle mass, were also altered in old rats compared with adult rats: Relative torque was lower at the highest stimulation frequencies (100 to 200 Hz; *P* < 0.01) (*Figure* S1). Consequently, maximal and mean relative torque were significantly lower in old than adult rats. Similar results were observed in dynamic conditions: Absolute power was reduced at all velocities, 50°/s excepted (*Figure* 1D–F), resulting in significantly lower maximal and mean power in old rats compared with adult rats. When expressed relative to muscle mass, power was reduced at contraction velocities ranging from 200 (*P* < 0.01) to 1000°/s (*P* < 0.001; *Figure* S1). Consequently, maximal and mean relative power were lower in old compared with adult rats. In addition, the maximal power was produced at a significantly lower optimal velocity in old than adult rats (475 ± 128 vs. 618 ± 98.2°/s; *P* = 0.01).

**Figure 2:**
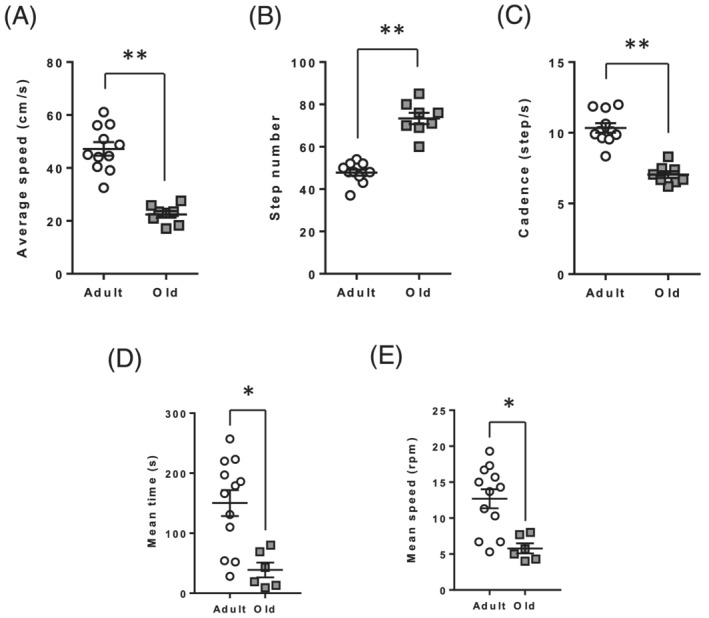
Locomotor activity and muscle coordination are impaired in old rats. Mobility and muscle coordination of adult and old rats were measured using catwalk (*A*–*C*) and rotarod (*D* & *E*), respectively. (*A*) Average speed to travel the catwalk tunnel. (*B*) Number of steps to complete the catwalk test. (*C*) Cadence to travel the catwalk tunnel. (*D*) Time spent on revolving rod in rotarod. (*E*) Mean speed reached by adult and old rats in the rotarod. Values are expressed as mean ± SEM. *P* values were assessed by unpaired *t*‐test. *P < 0.05, **P < 0.01 vs. adult.

We then studied mobility of adult and old rats. Voluntary activity, motor coordination, and quality of gait were analysed by using the open field, rotarod, and catwalk tests, respectively. Main catwalk data are summarized in *Figure*s 2 and S2. Old rats travelled the catwalk tunnel with a reduced average speed (*Figure* 2A, *P* < 0.01), complete the test with a higher number of steps (*Figure* 2B, *P* < 0.01), and a reduced cadence (*Figure* 2C, *P* < 0.01) compared with adult rats. This loss of mobility was associated with an impaired motor coordination as measured by the rotarod test (*Figure* 2D–E) and a decreased voluntary activity measured in the open field test (*Figure* S3).

### Analysis of endocannabinoids levels in plasma and tissues

To analyse whether sarcopenia was associated with alterations of the ECS, we first measured the levels of endocannabinoids, AEA and 2‐AG, and endocannabinoid‐like molecules, PEA and OEA in plasma, skeletal muscles, and adipose tissues. In plasma, AEA levels were similar between adult and old rats (*Table* 2). 2‐AG plasma levels were increased, yet not statistically, by ≈30% in old rats compared with adult rats (*Table* 2, *P* = 0.07). Plasma levels of PEA and OEA were reduced by ≈25% and ≈40%, respectively, in old rats compared with adult rats (*Table* 2, *P* < 0.01). We then measured endocannabinoid concentrations in soleus (oxidative) and EDL (glycolytic) skeletal muscles, and in gonadal (GWAT) and subcutaneous white adipose tissues (SWAT). In both soleus and EDL skeletal muscles, AEA and PEA contents were similar between adult and old rats (*Table* 2). However, we observed that soleus and EDL skeletal muscles showed distinct patterns in terms of 2‐AG and OEA contents: In the EDL, 2‐AG contents were similar between adult and old rats, whereas they were four‐fold higher in the soleus of old rats compared with adults (*Table* 2, *P* < 0.01). Conversely, in the soleus muscle, OEA contents were unchanged between adult and old rats, whereas they were reduced by ≈80% in the EDL of old rats compared with adult rats (*Table* 2, *P* < 0.01). In GWAT, the concentrations of AEA, 2‐AG, PEA, and OEA were similar in old rats compared with adults (*Table* 2). In SWAT, 2‐AG levels were unaltered between adult and old rats, whereas AEA, PEA, and OEA contents were all significantly increased in old compared with adult rats (*Table* 2).

### Endocannabinoid system receptor and enzyme expression

**Figure 3:**
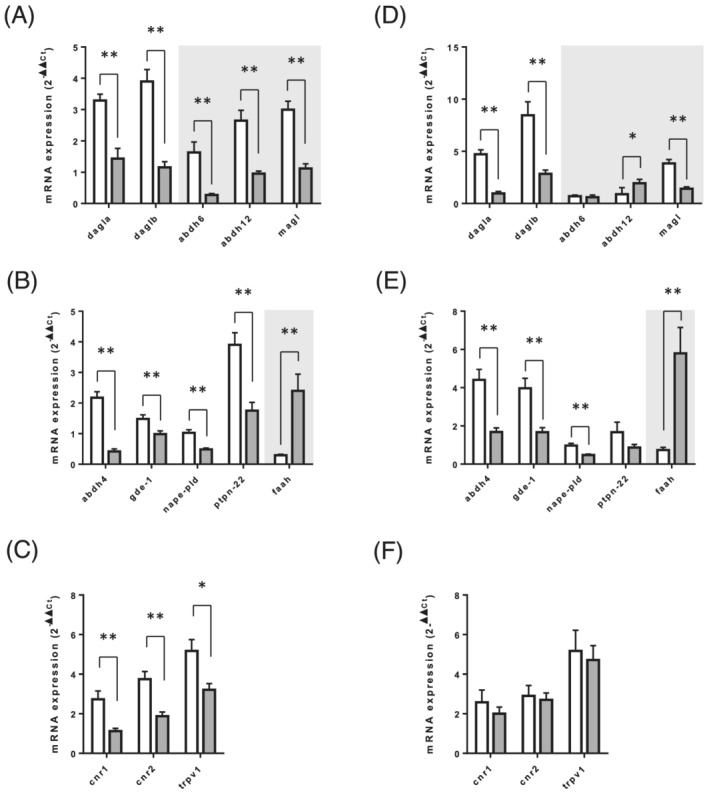
Expression level analysis of the genes related to endocannabinoid metabolism and function in skeletal muscle of adult and old rats. mRNA expression levels of genes encoding for (*A* & *D*) enzymes involved in 2‐AG biosynthesis (dagla & daglb) and catabolism (abhd6, abhd12, & magl), (*B* & *E*) enzymes involved in AEA and AEA congener biosynthesis (abhd4, gde‐1, nape‐pld, & ptpn22) and catabolism (faah), and (*C* & *F*) endocannabinoid receptors (cnr1, cnr2, & trpv1), were measured in soleus (*A–C*) and EDL (*D*–*F*) muscles. Values are expressed as mean ± SEM. *P* values were assessed by unpaired *t*‐test. *P < 0.05, **P < 0.01 vs. adult.

**Figure 4:**
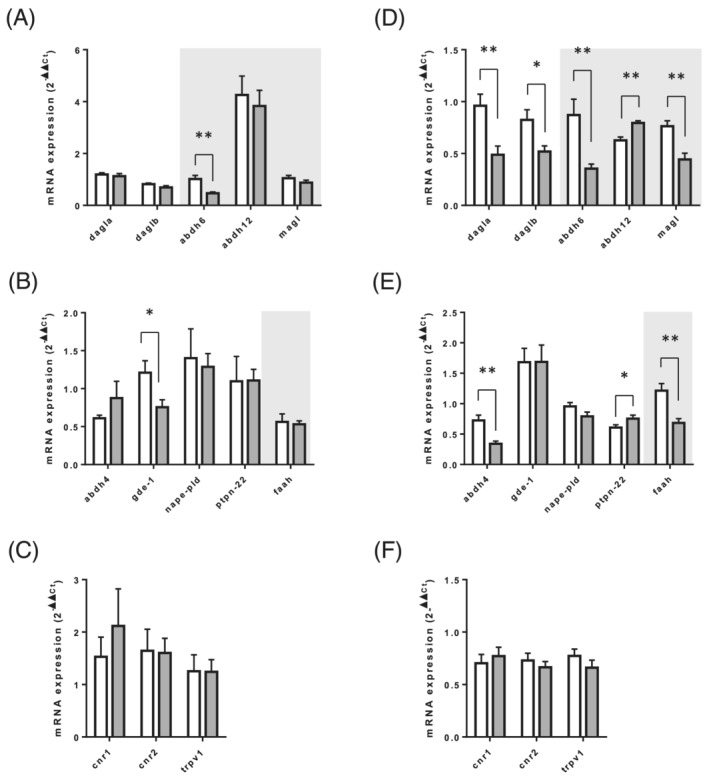
Expression level analysis of the genes related to endocannabinoid metabolism and function in adipose tissue of adult and old rats. mRNA expression levels of genes encoding for (*A*, *D*) enzymes involved in 2‐AG biosynthesis (dagla & daglb) and catabolism (abhd6, abhd12, & magl), (*B* & *E*) enzymes involved in AEA and AEA congener biosynthesis (abhd4, gde‐1, nape‐pld, & ptpn22) and catabolism (faah), and (*C* & *F*) endocannabinoid receptors (cnr1, cnr2, & trpv1), were measured in gonadal white adipose tissue (*A*–*C*) and subcutaneous adipose tissue (*D*–*F*) muscles. Values are expressed as mean ± SEM. *P* values were assessed by unpaired *t*‐test. *P < 0.05, **P < 0.01 vs. adult.

The mRNA expression of genes involved in the synthesis and degradation of endocannabinoids and endocannabinoid‐like molecules, as well as of genes encoding receptors involved in endocannabinoid signalling, were quantified by RT‐qPCR. As shown in *Figure* 3A and 3D, the expression of genes involved in the enzymatic system required for 2‐AG synthesis (DAGLα and DAGLβ) and degradation (ABDH6, ABDH12, and MAGL) were all drastically reduced in the soleus and EDL skeletal muscles of old compared with adult rats. The expression of the AEA and NAE biosynthesis machinery (ABDH4, GDE‐1, NAPE‐PLD, and PTPN22) was reduced (*Figure* 3B and 3E), whereas the expression of the AEA and NAE‐degrading enzyme FAAH was strongly increased, in both the soleus and EDL skeletal muscles of old rats (*Figure* 3B and 3E). Concerning the expression of genes encoding for endocannabinoid receptors, we observed that while the expression of CB1, CB2, and TRPV1 was unaltered in the EDL between old and adult rats, it was reduced in the soleus of old rats compared with adult rats (*Figure* 3C and 3F). We then measured the expression of the enzymes and receptors of the ECS in the adipose tissue. In the GWAT, only the expressions of ABDH6 and GDE‐1 transcripts were reduced in old rats compared with adults (*Figure* 4A–B). Unlike the GWAT, the expression of the ECS in the SWAT was more strongly altered in old rats. The expression of 2‐AG synthesis (DAGLα and DAGLβ) and degradation (ABDH6 and MAGL) enzymes were reduced in old rats compared with adults (*Figure* 4D), whereas ABDH12 expression was increased (*Figure* 4D). Concerning the AEA biosynthesis and degradation machinery in the SWAT, we observed a reduced expression of ABDH4 and FAAH and an increased expression of PTPN22 in old rats compared with adult ones (*Figure* 4E). The mRNA expression of CB1, CB2, and TRPV1 were unchanged between old and adult rats in both adipose tissues (*Figure* 4C and 4F). To further analyse whether some of these alterations in mRNA expression were also reflected at the protein level, we performed western blot analysis of CB1, MAGL, and FAAH proteins in the soleus, GWAT, and SWAT (*Figure* S4). CB1 protein expression was unaltered in the GWAT and SWAT and was reduced by ≈55% (*P* = 0.08) in old rat soleus compared with adults (*Figure* S4A–D). Western blot analysis also revealed a three‐fold increase in FAAH protein levels in old rat soleus (*P* < 0.01) and a ≈30% reduction in the SWAT as compared with adults (*P* < 0.05) (*Figure* S4A–C and S4E). Last, MAGL protein levels were unchanged in the GWAT, but reduced by ≈40% and ≈35% in the soleus (*P* < 0.05) and SWAT, respectively (*P* = 0.08) in old rats compared with adults (*Figure* S4A–C and S4F). These findings support and complement the observed alterations in MAGL, FAAH, and CB1 mRNA expression, described above.

### Correlation studies

**Figure 5:**
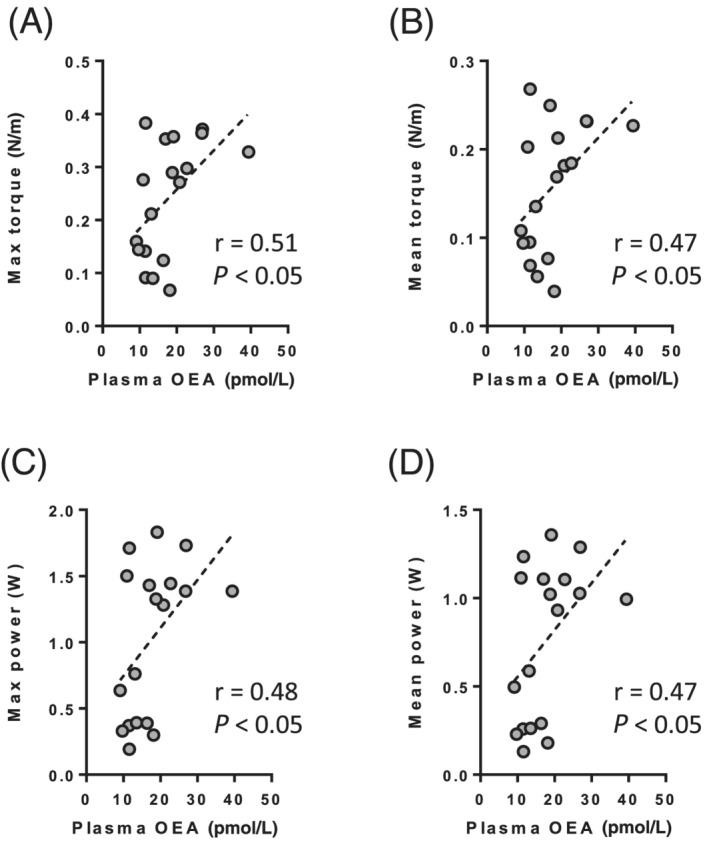
Pearson correlations analysis between plasma OEA levels and contractile properties of the plantarflexor muscles in rats. (*A*) Maximal absolute torque. (*B*) Mean absolute torque. (*C*) Maximal absolute power. (*D*) Mean absolute power. The graphs show the individual data points. OEA, oleoylethanolamide.

**Figure 6:**
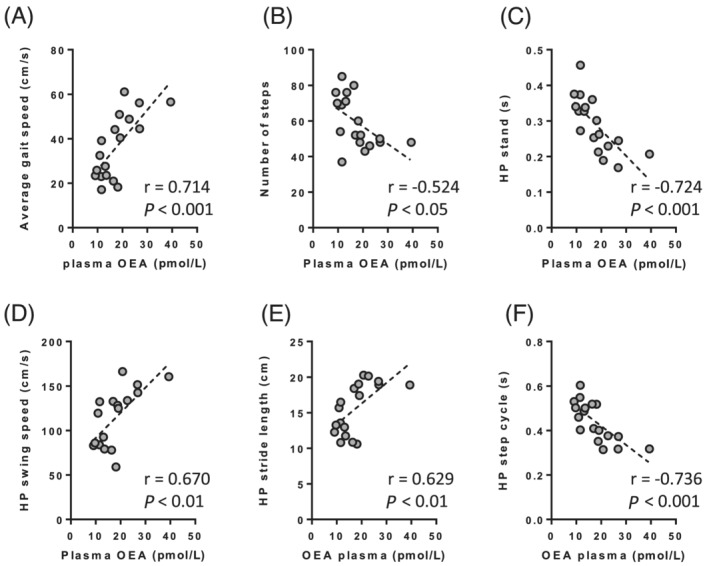
Pearson correlations analysis between plasma OEA levels and catwalk parameter of gait quality in rats. (*A*) Average speed to travel the catwalk tunnel. (*B*) Number of steps to complete the catwalk test. (*C*) Duration of rat hindlimb paw contact with the glass plate. (*D*) Time duration without rat hindlimb paw contact with the glass platform. (*E*) Distance between two placements of the same hindlimb foot. (*F*) Time between two consecutive initial contacts of the same hindlimb foot. The graphs show the individual data points. HP, hindlimb paw; OEA, oleoylethanolamide.

Correlation studies were performed to analyse whether endocannabinoid and endocannabinoid‐like molecule levels in plasma can be associated with body composition, and skeletal muscle mass and function. We observed that AEA and PEA plasma levels were not correlated with % fat mass, % lean mass, or hindlimb skeletal muscle mass (data not shown). 2‐AG plasma levels were positively correlated with % fat mass (*r* = 0.43, *P* = 0.08) and negatively correlated with % lean mass (*r* = −0.45, *P* = 0.06). OEA plasma levels were negatively correlated with % fat mass (*r* = −0.43, *P* = 0.07), and positively correlated with % lean mass (*r* = 0.48, *P* < 0.05) and with hindlimb skeletal muscle mass (*r* = 0.46, *P* = 0.06). We then correlated AEA, PEA, OEA, and 2‐AG plasma levels with the different parameters of muscle contractile properties and function. No correlation was found for AEA and 2‐AG plasma levels (data not shown) except for AEA plasma levels that were negatively correlated with optimal velocity (*r* = −0.50, *P* < 0.05). OEA plasma levels were positively correlated with absolute values of maximal torque (*r* = 0.51, *P* < 0.05), mean torque (*r* = 0.47, *P* < 0.05), maximal power (*r* = 0.48, *P* < 0.05), and mean power (*r* = 0.47, *P* < 0.05) (*Figure* 5). However, muscle mass cannot fully account for these correlations since OEA plasma levels were still positively correlated with maximal torque (*r* = 0.46, *P* = 0.053) and power (*r* = 0.47, *P* < 0.05) when expressed relative to muscle mass (*Figure* S5). Although non‐significant, similar results were observed for mean relative torque (*r* = 0.35, *P* = 0.16) and power (*r* = 0.44, *P* = 0.06) (*Figure* S5). OEA, and to a lesser extent PEA (data not shown), plasma levels were significantly correlated with the average gait speed (*Figure* 6A), the number of steps to complete the test (*Figure* 6B), and all the different hindlimb paw parameters analysed in the catwalk test (stand, swing speed stride length, and step cycle, *Figure* 6C–F). Similar correlations between OEA and voluntary activity and motor coordination were observed in the open field and rotarod tests (*Figure* S6).

## Discussion

The first aim of this study was to examine whether the circulating and tissue levels of endocannabinoids (AEA & 2‐AG) and of two endocannabinoid‐like molecules (PEA & OEA) were altered with sarcopenia and to characterize the effect of sarcopenia on the expression of genes encoding for the enzymatic machinery regulating the synthesis and degradation of these mediators and for endocannabinoid receptors in the adipose tissue and skeletal muscle of adult and old rats. The second aim was to explore whether alterations of the ECS can be associated with skeletal muscle dysfunction and loss of mobility. In summary, we demonstrated that sarcopenia is associated with (1) reduced PEA and OEA circulating levels; (2) distinct alterations in 2‐AG and OEA muscle levels, according to the muscle typology, for example, in EDL and in soleus; (3) an increased accumulation of NAEs in the GWAT; and (4) profound modifications in the expression of the ECS genes in the GWAT and skeletal muscles. Last we observed that OEA, and to a less extent PEA, plasma levels, were correlated with muscle mass and function in rats.

The significant correlations observed between OEA plasma levels and muscle contractile properties measured with the force–frequency and power–velocity relationships are a matter of debate. On the one hand, it has been demonstrated that endocannabinoids, and particularly AEA, are able to modulate contractile activity at two distinct levels.^18^ At the neuromuscular junction, these molecules are able to adjust the parameters for quantal acetylcholine release from the motor nerve terminal.^19^, ^20^ In addition, endocannabinoids modulates the excitation–contraction coupling by limiting the depolarization‐induced Ca^2+^ release from the sarcoplasmic reticulum.^21^ The reduced expression of the NAE biosynthesis machinery, and the increased expression of the NAE‐degrading enzyme FAAH, observed in the current study in old rats could thus be consistent with (1) the observed relative preservation of force production at low stimulation frequencies, indicative of preserved excitation–contraction coupling, and (2) the selective depression of force at the highest stimulation frequencies, potentially indicative of impaired modulation of neuromuscular transmission. However, AEA levels were not altered in the soleus muscle, and no correlation was observed between AEA plasma levels and contractile properties, except for the optimal velocity. OEA levels were instead reduced in the EDL of old rats, an effect that is likely due to reduction and increase in the expression of NAE biosynthetic and degrading enzymes, respectively.

Significant positive correlations were observed between OEA plasma levels and contractile and functional properties. These correlations were first observed with absolute torque and power values and parameters from the locomotion and activity tests. As OEA was also correlated with the percentage of lean mass, one may argue that these correlations simply reflect the fact that old rats have a reduced muscle mass and therefore, release a smaller amount of OEA in the bloodstream. However, some correlations were still observed when expressing torque and power relative to muscle mass. Interestingly, plasma levels of endocannabinoids and OEA have been shown to increase following exercise intensity.^17^, ^22^ The reduced physical activity levels of old rats may then have contributed to a reduced production of endocannabinoid‐like molecules and consequently, to the observed reduced OEA plasma levels, independently of muscle mass. However, direct evidence is lacking to support these various hypotheses and the relationships between endocannabinoids, muscle contractile properties, and the resulting functional properties and motor activity. Additional studies are thus required to determine whether the depression of the ECS reported in old rats is the cause or consequence of the observed altered muscle size, properties, and functional abilities. Exercise activity, as a modulator of endocannabinoid production and skeletal muscle size and contractile properties, could be an interesting paradigm to get insight into these mechanisms.

Sarcopenia and obesity share common alterations leading to muscle impairment (such as insulin resistance, increased adiposity, and muscle lipotoxicity), and it is well known that the peripheral ECS is overactive in response to obesity.^8^ To our knowledge, the present study is the first to characterize the alterations of the ECS in the context of sarcopenia. In the skeletal muscle, AEA levels were increased in the soleus of obese Zucker^fa/fa^ rats as compared to lean Zucker^fa/+^ rats, and these changes were associated with increased *Abdh4*, *gde‐1* and *faah* mRNA expression.^23^ Similarly, 24 weeks of high‐fat diet increased AEA levels in both soleus and EDL, in line with reduced *faah* mRNA and increased *abdh4* mRNA expressions.^17^ Data for 2‐AG are conflicting with results showing decrease^17^ or no changes^23^ in soleus 2‐AG contents. In these two previous studies, OEA and PEA levels were unaltered by obesity. In the present study, we documented an increased 2‐AG content in the soleus and a reduced OEA content in EDL. As mentioned above, the reduced OEA levels was probably due to the reduced expression of NAE biosynthesis enzymes (*Abdh4*, *Gde‐1*, *Nape‐pld*, and *Ptpn22*) and/or increased expression of the NAE‐degrading enzyme, *Faah*. However, these transcriptional effects were observed also in the soleus, and therefore, it is not clear why OEA levels did not decrease also in this tissue, nor why AEA content was decreased in the skeletal muscle only to a much lesser, and statistically non‐significant, extent. In the case of 2‐AG, its increase in the soleus could be due to decreased expression of its degrading enzymes (*Abhd6*, *Abdh12*, and *Mgll*), but not to changes in its biosynthetic enzymes (*Dagla* & *Daglb*), which were also decreased; again, such changes also occurred in the EDL where 2‐AG levels were not altered. Indeed, the tissue levels of endocannabinoids and their NAE and MAG congeners are also regulated by the availability of their ultimate fatty acid biosynthetic precursors (e.g. arachidonic acid and oleic acid for AEA and OEA, respectively).^24^ It is therefore conceivable that, under certain conditions, precursor availability might have a stronger impact on tissue endocannabinoid concentrations than enzyme expression.

Increased 2‐AG contents in soleus are likely to cause overactivation of CB1, which is associated with the loss of muscle mass and function, and of mitochondrial activity in oxidative soleus during aging. Indeed, CB1 is widely distributed in the brain and peripheral organs where it regulates cellular functions and metabolism.^8^ It is well known that ECS activity modulates peripheral tissue and, more specifically, skeletal muscle insulin sensitivity. *In vitro*, the treatment of skeletal muscle cells or muscle explants with adipocyte‐conditioned medium containing AEA and 2‐AG, or with synthetic CB1 agonists, significantly alters glucose uptake and insulin sensitivity, in part by inhibiting Akt activation.^10^, ^12^ Therefore, the increased 2‐AG content in soleus might be associated with reduced insulin sensitivity and lead to reduced anabolic response. CB1 is also localized on skeletal muscle mitochondria where it directly controls cellular respiration and ATP production,^25^ and its activation is known to impair mitochondrial function and biogenesis.^25^, ^26^, ^27^, ^28^, ^29^ This process is in part mediated through the production of ceramides,^29^ lipid derivatives associated with the development of lipotoxicity and insulin resistance commonly seen in sarcopenia.^5^ Lastly, CB1 overactivation is also associated with skeletal muscle development and function. Iannotti *et al*. demonstrated that 2‐AG levels decreased both during myotube formation in C2C12 myoblasts and during mouse muscle growth *in vivo*.^13^ Furthermore, *in vitro*, CB1 stimulation by 2‐AG or ACEA was able to prevent myotube formation from human and mouse myoblasts.^13^ Thus, the increased levels of 2‐AG found here in the soleus might underlie some of the pathological features of the skeletal muscle in aged rats.

Reduced OEA content in EDL might affect muscle metabolism and development. Indeed, OEA exhibits anti‐inflammatory effects and modulates lipid metabolism^9^ mainly through PPARα.^30^ A second receptor target of OEA is TRPV1, a capsaicin‐sensitive receptor that is expressed in numerous tissues, including skeletal muscle.^31^ We observed that, although TRPV1 expression was unaltered in the EDL between old and adult rats, it was reduced in the soleus of old rats compared with adult rats. Interestingly, several studies delineate the role of TRPV1 in skeletal muscle metabolism and development. TRPV1 is known to regulate glucose uptake into skeletal muscle.^32^ Acute administration of capsaicin enhances fatty acid utilization and endurance capacity in rodents.^33^ Similarly, long‐term activation of TRPV1 by capsaicin or overexpression improves exercise endurance and energy metabolism in mice by stimulating fatty acid oxidation and mitochondrial biogenesis, through increased expression of PGC1α.^34^ Furthermore, in a model of overload‐induced hypertrophy, Ito *et al*. observed that skeletal muscle hypertrophy was associated with an increased TRPV1 activity and that capsaicin administration was sufficient to induce skeletal muscle hypertrophy.^35^ This is in line with the ability of TRPV1 to modulate molecular processes regulating muscle differentiation and repair. Indeed, TRPV1 activation by heat induces the expression of myogenic transcription factors in myoblasts,^36^ and capsaicin facilitates muscle repair after cardiotoxin‐induced muscle injury.^37^ Some of these functions have been described also for PPARα, whose decreased expression (possibly explained by decreased levels of its endogenous ligands) has been associated with sarcopenia.^38^

An increase in NAE (AEA, OEA, and PEA), but not 2‐AG, levels was found here in the SWAT of aged rats (although plasma 2‐AG levels positively correlated with fat mass, in agreement with previous studies^39^). This finding could be explained by the concomitant decrease in the expression of *Faah* and increase in the expression of *Ptnp22*, which may have caused decreased degradation and, possibly, increased biosynthesis of NAEs, and by the simultaneous increase in the expression of both biosynthetic and catabolic enzymes for 2‐AG in this tissue. Increased AEA levels, and, hence, stronger activation of CB1 with prolipogenic and proadipogenic activity,^39^ may underlie the increased fat mass at the expense of lean mass in aged rats. Conversely, increased levels of OEA and PEA (two endogenous PPARα agonist with strong anti‐inflammatory activity^30^) may instead represent an adaptive response aimed at counteracting inflammation in sarcopenia.

In summary, our present results indicate that the response of the ECS and of endocannabinoid‐like mediators to aging depends on skeletal muscle type (i.e. glycolytic vs. oxidative). Therefore, with sarcopenia, some of the alterations of the ECS might be differently orchestrated in oxidative and glycolytic skeletal muscles: On the one hand, the reduced OEA levels observed in the EDL could result in a reduced TRPV1 and PPARα activity leading to an impairment of mitochondrial biogenesis and function and of skeletal muscle regeneration. On the other hand, the increased soleus 2‐AG levels might, via excessive CB1 activity, alter muscle regeneration, contractile activity, and insulin sensitivity in a way leading to anabolic resistance, and also impinge mitochondrial function and biogenesis, processes that are highly affected in sarcopenia. Further studies, using both pharmacological and genetic tools to manipulate the several receptors involved in endocannabinoid and OEA and PEA action, are now needed to fully dissect the role of these mediators and their molecular targets in age‐induced sarcopenia. Also, the modifications of circulating ECs and their putative correlation with muscle function that we documented in a rat model of sarcopenia should be addressed and confirmed in human cohorts.

## Conflict of interest

The authors declare they have no conflict of interest.

## Funding

This study was supported by the French government IDEX‐ISITE initiative 16‐IDEX‐0001 (CAP 20‐25) and I‐SITE project (CAP 2025) of the University of Clermont Auvergne, and the Joint International Research Unit on Chemical and Biomolecular Studies of the Microbiome in Metabolic Health, which is in turn supported by the Sentinelle Nord project of Université Laval and the Consiglio Nazionale delle Ricerche of Italy.

## Supporting information


**Table S1.** Primer sequences used in qPCR analysis
**Table S2.** Adult and old rat metabolic parameters
**Figure S1.** Contractile properties of the plantarflexor are impaired in old rats. A) Relative torque‐frequency relationship in adult and old rats. B) Maximal relative torque. C) Mean relative torque. D) Relative power‐frequency relationship in adult and old rats. E) Maximal relative power. F) Mean relative power. Values are expressed as mean ± sem. p values were assessed by two‐way ANOVA and Tukey post‐test as described in Materials and Methods (in A, D), or by unpaired t‐test (in B, C, E, F). ** *p* < 0.01 vs. adult.
**Figure S2.** Locomotor activity of adult and old rats measured using catwalk. A) Duration of rat paw contact with the glass plate. B) Time duration without rat paw contact with the glass platform. C) Distance between two placements of the same foot. D) Time between two consecutive initial contacts of the same foot. Values are expressed as mean ± sem. *p* values were assessed by unpaired t‐test. HP: Hindlimb paw, FP: front paw. ** *p* < 0.01 vs. adult.
**Figure S3.** Locomotor activity of adult and old rats measured using openfield. A) Exploratory locomotor activity as measured by total travelled distance over a 10‐min test period. B) Average speed over a 10‐min test period. C) Duration of activity over a 10‐min test period. Values are expressed as mean ± sem. *p* values were assessed by unpaired t‐test. HP: Hindlimb paw, FP: front paw. ** *p* < 0.01 vs. adult.
**Figure S4.** CB1, FAAH and MAGL protein level expression in adult and old rat tissues. Representative western blot of CB1, FAAH and MAGL protein levels in soleus (A), GWAT (B) and SWAT (C) are shown. Quantification of CB1 (D), FAAH (E) and MAGL (F) signals from A, B and C. Results are expressed as mean ± SEM. * *p* < 0.05, ** *p* < 0.01 versus adult.
**Figure S5.** Pearson correlations analysis between plasma OEA levels and contractile properties of the plantarflexor muscles in rats. A) Maximal relative torque. B) Mean relative torque. C) Maximal relative power. D) Mean relative power. The graphs show the individual data points.
**Figure S6.** Pearson correlations analysis between plasma OEA levels and locomotor activity measured using openfield (A‐C) and motor coordination measured using rotarod (D, E) of adult and old rats. A) Total travelled distance over a 10‐min test period. B) Average speed over a 10‐min test period. C) Duration of activity over a 10‐min test period. D) Time spent until the rat fell from the rotarod. E) Speed achieved when the rat fell rotarod. The graphs show the individual data points.
