# Supplementary material for: Alterations of the endocannabinoid system and circulating and peripheral tissue levels of endocannabinoids in sarcopenic rats
Source: J Cachexia Sarcopenia Muscle. 2021 Dec 2;13(1):662–76. doi: 10.1002/jcsm.12855 (PMC8818601; doi:10.1002/jcsm.12855)
Supplement: Supplementary file 1 — Table S1. Primer sequences used in qPCR analysis Table S2. Adult and old rat metabolic parameters Figure S1. Contractile properties of the plantarflexor are impaired in old rats. A) Relative torque‐frequency relationship in adult and old rats. B) Maximal relative torque. C) Mean relative torque. D) Relative power‐frequency relationship in adult and old rats. E) Maximal relative power. F) Mean relative power. Values are expressed as mean ± sem. p values were assessed by two‐way ANOVA and Tukey post‐test as described in Materials and Methods (in A, D), or by unpaired t‐test (in B, C, E, F). ** p < 0.01 vs. adult. Figure S2. Locomotor activity of adult and old rats measured using catwalk. A) Duration of rat paw contact with the glass plate. B) Time duration without rat paw contact with the glass platform. C) Distance between two placements of the same foot. D) Time between two consecutive initial contacts of the same foot. Values are expressed as mean ± sem. p values were assessed by unpaired t‐test. HP: Hindlimb paw, FP: front paw. ** p < 0.01 vs. adult. Figure S3. Locomotor activity of adult and old rats measured using openfield. A) Exploratory locomotor activity as measured by total travelled distance over a 10‐min test period. B) Average speed over a 10‐min test period. C) Duration of activity over a 10‐min test period. Values are expressed as mean ± sem. p values were assessed by unpaired t‐test. HP: Hindlimb paw, FP: front paw. ** p < 0.01 vs. adult. Figure S4. CB1, FAAH and MAGL protein level expression in adult and old rat tissues. Representative western blot of CB1, FAAH and MAGL protein levels in soleus (A), GWAT (B) and SWAT (C) are shown. Quantification of CB1 (D), FAAH (E) and MAGL (F) signals from A, B and C. Results are expressed as mean ± SEM. * p < 0.05, ** p < 0.01 versus adult. Figure S5. Pearson correlations analysis between plasma OEA levels and contractile properties of the plantarflexor muscles in rats. A) Maximal relative torque. B) Mean relativ [file JCSM-13-662-s001.pdf]

## SUPPLEMENTAL DATA

**Supplemental Table 1.** Primer sequences used in qPCR analysis

| Gene | Forward sequence | Reverse sequence |
|---|---|---|
| Abdh12 | 5’-CAGGCGTGCGGTCGAAACCA-3’ | 5’-TCAAGCTGCAGTCGGCGTCC-3’ |
| Abdh4 | 5’-TCTGGCGTCAAGCGGAGGGA-3’ | 5’-ACGCCACCCCCAAAGCCATG-3’ |
| Abdh6 | 5’-AGCGTCTGCTCCCATCCCCA-3’ | 5’-TGGCTTGCCAGTGGCGTGAA-3’ |
| Cnr1 | 5’-CTGAGGGTTCCCTCCCGGCA-3’ | 5’-TGCTGGGACCAACGGGGAGT-3’ |
| Cnr2 | 5’-AAGCCCCAAAGTCCTCAGTT-3’ | 5’-CCCAACTCCTGCTTATCCTTC-3’ |
| Dagla | 5’-GGCCGCACCTTCGTCAAGCT-3’ | 5’-ATCCAGCACCGCATTGCGCT-3’ |
| Daglb | 5’-AGACCCGGGTGCAATGCTGC-3’ | 5’-GCCCTGGTGTGTGGGTCACG-3’ |
| Faah | 5’-GGCAGAGCCACAGGGGCTATCA-3’ | 5’-TGGGGCTACAGTGCACAGCG-3’ |
| Hprt | 5’-TTGCTGACCTGCTGGATTAC-3’ | 5’-AGTTGAGAGATCATCTCCAC-3’ |
| Gde1 | 5’-GCAGCCCCTTCAACGCCTGT-3’ | 5’-GATGGCCGCCAGCGTGTTCT-3’ |
| Magl | 5’-CGGAACAAGTCGGAGGTTGA-3’ | 5’-TGTCCTGACTCGGGGATGAT-3’ |
| Nape-pld | 5’-AGGCTGGCCTACGAATCACGT-3’ | 5’-ATGGTACACGGGGGACGGCG-3’ |
| Ptpn22 | 5’-TGGTCGTGGGAGAGCCGCTT-3’ | 5’-GGGCCACTTTTTGCGCCTGC-3’ |
| Trpv1 | 5’-AGACATCAGCGCCCGGGACT-3’ | 5’-CCAGCTTCAGCGTGGGGTGG-3’ |
| Ubc | 5’-ACACCAAGAAGGTCAAACAGGA-3’ | 5’-CACCTCCCCATCAAACCCAA-3’ |

**Supplemental Table 2.** Adult and old rat metabolic parameters

| | **Adult** | **Old** |
|---|---|---|
| Insulin (ng/ml) | 0.79 ± 0.17 | 1.21 ± 0.22 |
| Glucose (g/l) | 1.39 ± 0.08 | 1.48 ± 0.07 |
| Cholesterol (g/l) | 0.87 ± 0.07 | 1.19 ± 0.08* |
| TAG (g/l) | 0.91 ± 0.13 | 0.74 ± 0.07 |
| NEFA (mg/l) | 164.7 ± 10.7 | 181.5 ± 18.2 |

Rats were 6-month (adults) or 24-month old. Blood parameters were measured after overnight fasting. Results are expressed as mean ± sem (n=8-11). P values were assessed by a with a Welch's two-sample unpaired *t*-test. * P<0.05, *vs.* Adult rats.

**Supplemental Figure S1.**

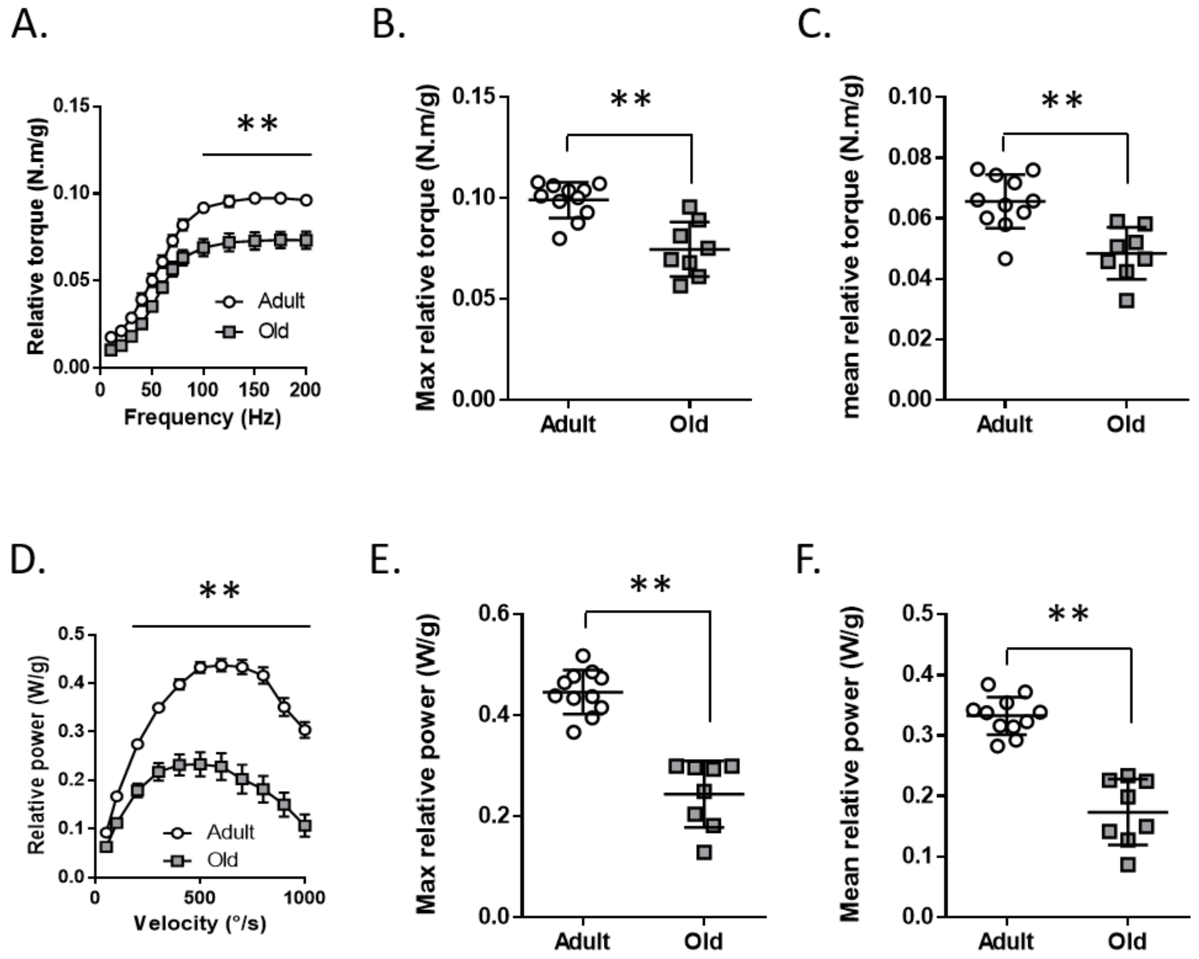


**Fig. S1** Contractile properties of the plantarflexor are impaired in old rats. A) Relative torque-frequency relationship in adult and old rats. B) Maximal relative torque. C) Mean relative torque. D) Relative power-frequency relationship in adult and old rats. E) Maximal relative power. F) Mean relative power. Values are expressed as mean ± sem. p values were assessed by two-way ANOVA and Tukey post-test as described in Materials and Methods (in A, D), or by unpaired t-test (in B, C, E, F). ** $p < 0.01$ vs. adult.

**Supplemental Figure S2.**

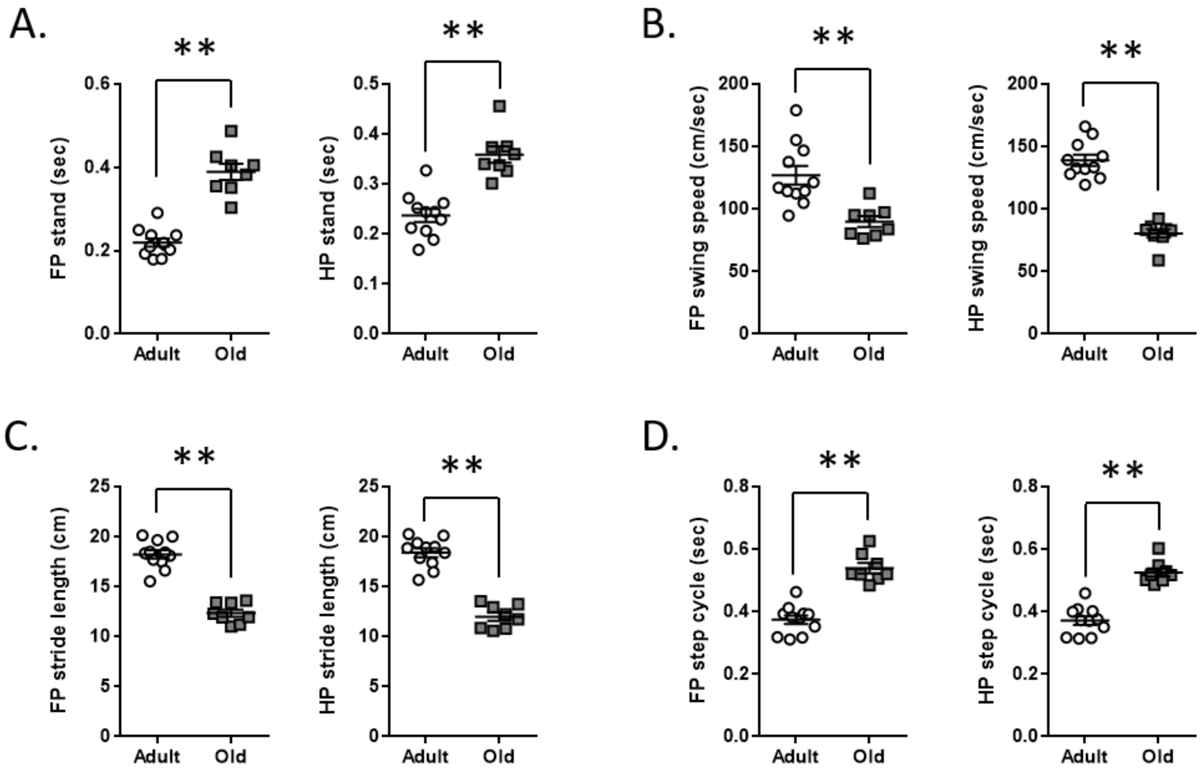


**Fig. S2** Locomotor activity of adult and old rats measured using catwalk. A) Duration of rat paw contact with the glass plate. B) Time duration without rat paw contact with the glass platform. C) Distance between two placements of the same foot. D) Time between two consecutive initial contacts of the same foot. Values are expressed as mean ± sem. *p* values were assessed by unpaired t-test. HP: Hindlimb paw, FP: front paw. ** $p < 0.01$ vs. adult.

**Supplemental Figure S3.**

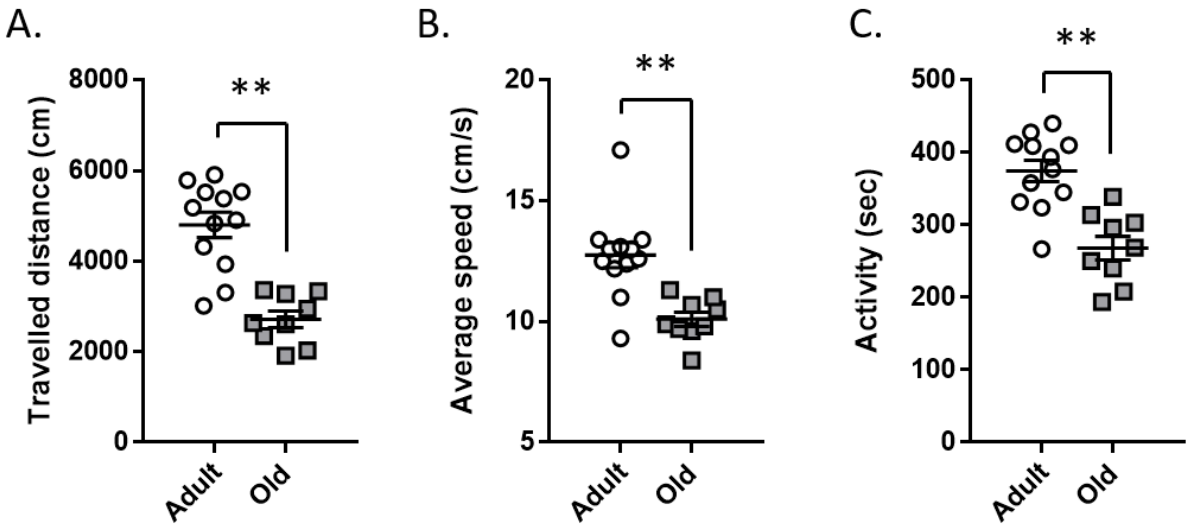


**Fig. S3** Locomotor activity of adult and old rats measured using openfield. A) Exploratory locomotor activity as measured by total travelled distance over a 10-min test period. B) Average speed over a 10-min test period. C) Duration of activity over a 10-min test period. Values are expressed as mean ± sem. *p* values were assessed by unpaired t-test. HP: Hindlimb paw, FP: front paw. ** $p < 0.01$ vs. adult.

**Supplemental Figure S4.**

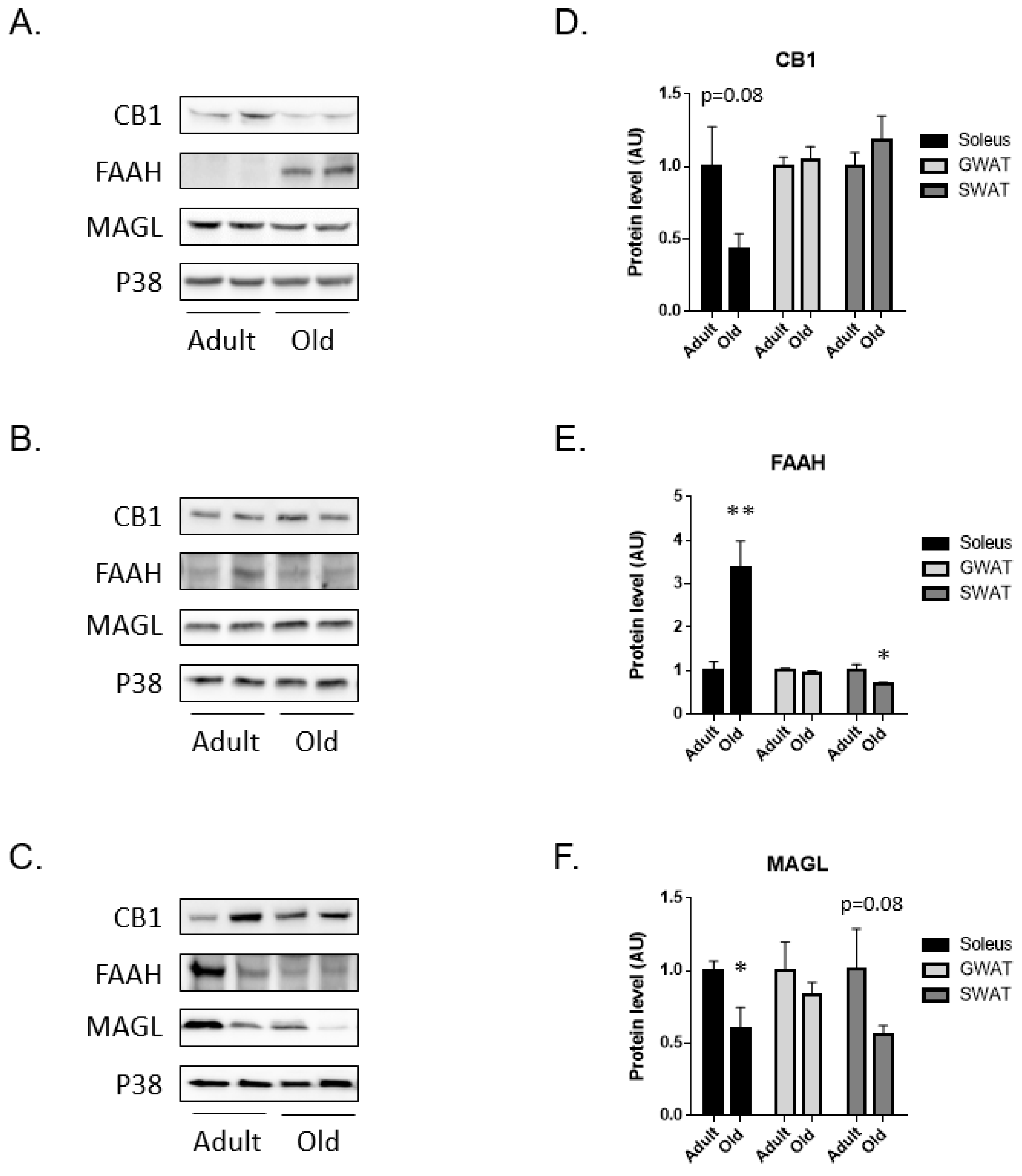


**Fig. S4** CB1, FAAH and MAGL protein level expression in adult and old rat tissues. Representative western blot of CB1, FAAH and MAGL protein levels in soleus (A), GWAT (B) and SWAT (C) are shown. Quantification of CB1 (D), FAAH (E) and MAGL (F) signals from A, B and C. Results are expressed as mean ± SEM. * $p < 0.05$, ** $p < 0.01$ versus adult.

**Supplemental Figure S5.**

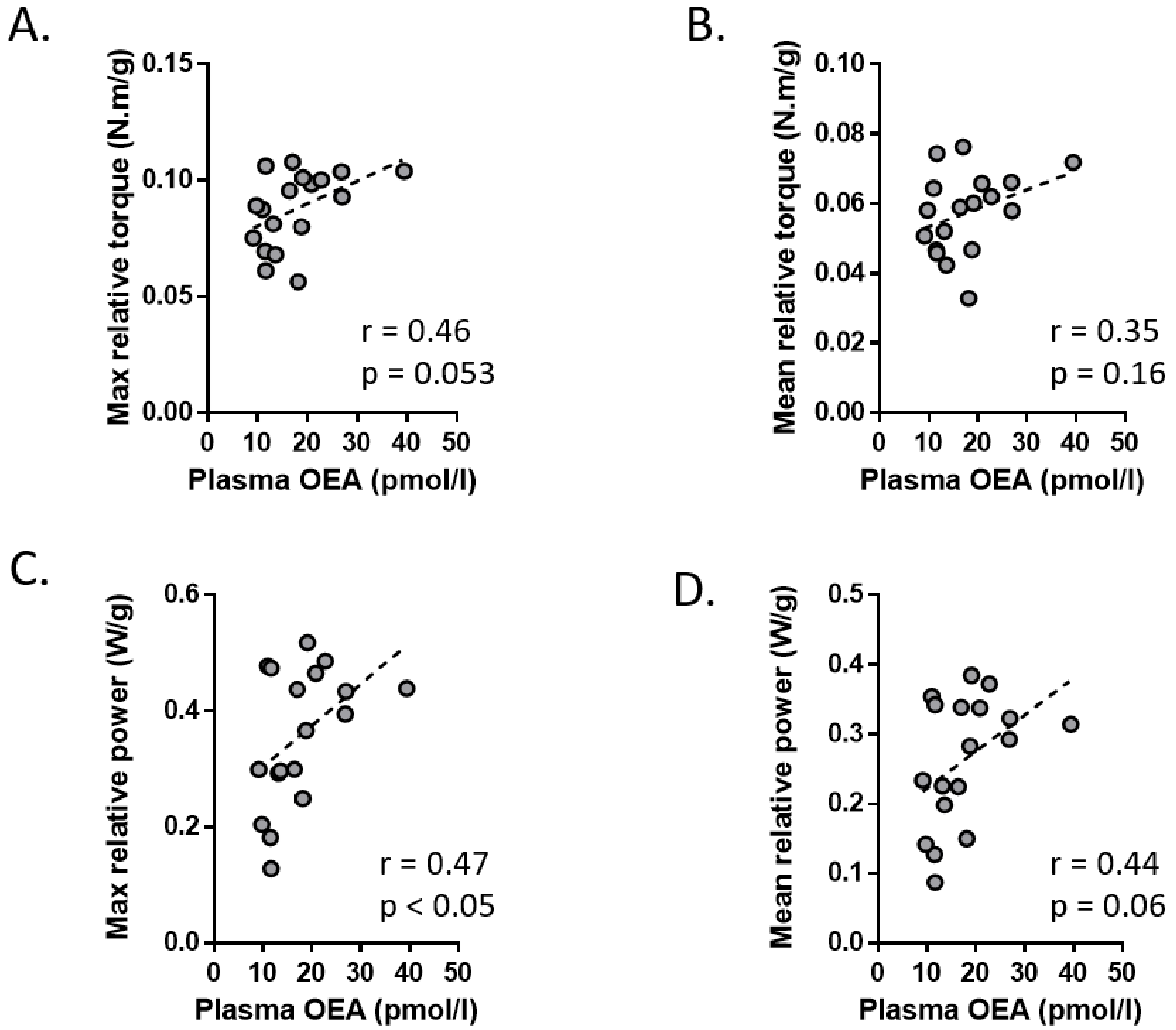


**Fig. S5** Pearson correlations analysis between plasma OEA levels and contractile properties of the plantarflexor muscles in rats. A) Maximal relative torque. B) Mean relative torque. C) Maximal relative power. D) Mean relative power. The graphs show the individual data points.

**Supplemental Figure S6.**

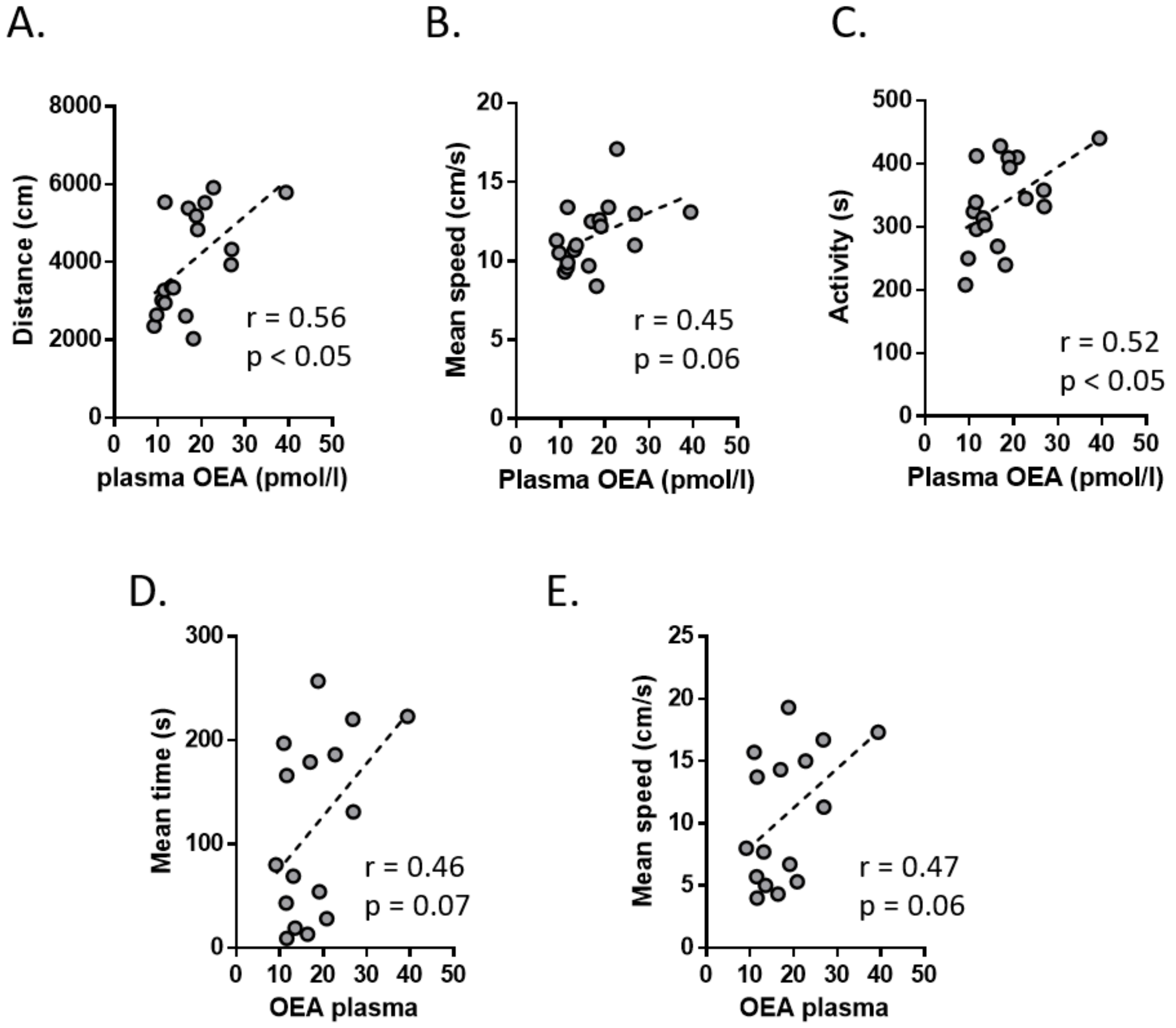


**Fig. S6** Pearson correlations analysis between plasma OEA levels and locomotor activity measured using openfield (A-C) and motor coordination measured using rotarod (D, E) of adult and old rats. A) Total travelled distance over a 10-min test period. B) Average speed over a 10-min test period. C) Duration of activity over a 10-min test period. D) Time spent until the rat fell from the rotarod. E) Speed achieved when the rat fell rotarod. The graphs show the individual data points.
